# Exploring the Palmar Surface: A Critical Case Report for Emergency Physicians

**DOI:** 10.5811/cpcem.6657

**Published:** 2024-09-06

**Authors:** Matthew Van Ligten, Douglas E. Rappaport, Wayne A. Martini

**Affiliations:** * Mayo Clinic Alix School of Medicine, Phoenix, Arizona; † Mayo Clinic Arizona, Department of Emergency Medicine, Phoenix, Arizona

**Keywords:** tendon injuries, flexor tendon rupture, emergency department, hand surgery, comorbidities, case report

## Abstract

**Introduction:**

Tendon injuries of the hand present a diverse spectrum of challenges in emergency medicine, ranging from minor strains to catastrophic ruptures. The superficial anatomy of hand tendons predisposes them to various mechanisms of injury, leading to complex medical scenarios. Here, we present a unique case of flexor tendon exposure secondary to abscess formation and spontaneous rupture, emphasizing the importance of prompt recognition and management of such injuries in the emergency department.

**Case Report:**

A 69-year-old male with multiple comorbidities presented with diffuse pain and a pale, pulseless right lower extremity, alongside a left hand exhibiting exposed flexor tendons due to recent abscess drainage. Despite broad-spectrum antibiotics and pain management, the patient underwent above-knee amputation due to vascular compromise. Evaluation revealed a complete flexor tendon rupture likely attributable to infection, necessitating emergent hand surgery at the bedside.

**Conclusion:**

Understanding the nuances of tendon injuries is paramount for emergency physicians, given their potential for lifelong disability if inadequately addressed. Awareness of risk factors and appropriate management strategies, including early surgical intervention when indicated, is essential in optimizing patient outcomes. This case serves as a reminder of the complexities involved in hand injuries and underscores the need for vigilance and tailored care in the emergency setting.

## INTRODUCTION

Tendon injuries are commonly seen in the emergency department (ED) with causes ranging from minor falls and lacerations to major trauma. The substantial number of mechanisms of injury to the hand coupled with the superficial anatomy of the tendons that allow movement leads to a broad range of medical complexities. Closed injuries such as the commonly seen “mallet finger” can be splinted to allow for healing, while complete tearing and/or exposure of the tendon may require surgical intervention to prevent lifelong disability.^1^ Moreover, while incomplete ruptures may allow the patient to remain functional, these injuries can evolve over time leading to a complete rupture later. Diagnoses of these injuries require a thorough physical exam to test for neurological function, imaging with radiographs to rule out bone abnormalities, and ultrasound and/or magnetic resonance imaging for definitive diagnosis of a soft tissue injury. Here, we present a unique presentation of a patient with flexor tendon exposure in the setting of recent abscess formation with spontaneous rupture.

## CASE REPORT

The patient was a 69-year-old, Russian-speaking male with a significant medical history including type 2 diabetes, peripheral arterial disease (PAD) status post multiple bilateral lower extremity revascularization procedures, coronary artery disease (CAD), hypertension, rheumatoid arthritis (RA), and recently diagnosed gastric adenocarcinoma. He presented to our ED with diffuse pain in his body, but primarily in his right leg and left hand.

He had been seen two days earlier at an outside ED where he had an abscess on the left hand that had spontaneously ruptured. At that time, he was treated with metronidazole, doxycycline, and trimethoprim-sulfamethoxazole.

**Image 1:**
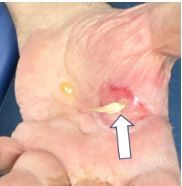
Photo of patient’s left hand with spontaneous tendon rupture (arrow) present as well as serosanguinous fluid present.

**Image 2:**
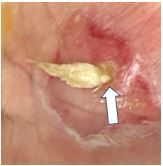
Zoomed-in view of patient’s left hand with spontaneous tendon rupture (arrow) present as well as serosanguinous fluid present.

On his evaluation in our ED, the patient was in distress secondary to his pain. The right lower extremity was identified as pale, cold, and pulseless. His left hand showed exposed shiny, ribbon-like substance protruding from the palmar aspect with serosanguinous fluid draining (Images 1 and 2). We attempted to maneuver the substance with 4x4 cotton, which revealed it to be anchored beneath the surface. Flexion of fingers and palm caused the substance to withdraw nearly entirely beneath the surface.

He was diagnosed with acute limb ischemia suspected to be secondary to an acute arterial occlusion, as well as spontaneous flexor tendon rupture secondary to an abscess within the left hand. He was started on broad spectrum antibiotics (as treatment for tendon infection and prophylaxis for the operating room), pain medication, and high-dose heparin drip. Emergent evaluation by vascular surgery and hand surgery teams were called to the ED for evaluation. Due to the vascular emergency of his right lower extremity, the patient was sent to vascular surgery for attempted salvation; however, he was deemed inoperable, which resulted in an above the knee amputation. Evaluation of his left hand showed a full tendon rupture, thought to be due to infection and spontaneous abscess drainage before arriving in the ED two days prior when the abscess was still in place. Due to his medical complications, the patient’s hand surgery was performed at bedside with irrigation and washout with consideration of further intervention in the future.

**CPC-EM Capsule**

What do we already know about this clinical entity?

*Tendon injuries in the hand vary from minor strains to catastrophic ruptures, necessitating prompt recognition and management due to potential lifelong disability.*

What makes this presentation of disease reportable?

*This rare case of flexor tendon exposure due to abscess and spontaneous rupture, highlights complexities in diagnosis and management.*

What is the major learning point?

*It is vital to recognize risk factors such as comorbidities (eg, rheumatoid arthritis, peripheral arterial disease), and the need for cautious intervention in hand pathologies, and timely surgical management.*

How might this improve emergency medicine practice?

*Emergency physicians should be aware of the nuanced presentations of hand injuries, facilitating tailored and timely interventions to optimize patient outcomes.*

## DISCUSSION

Emergency physicians need to promptly recognize and manage tendinous injuries due to their diverse presentation, severity, and treatment requirements. Initial treatment requires a thorough physical exam, focusing on neurological status and motor function. The treatment depends on the severity with most partial ruptures being treated conservatively. Some complete extensor tendon partial tears or ruptures, such as mallet and Jersey finger, can be treated in the ED with splinting and outpatient follow-up.^2^ Other clear indications for surgical management would be complete rupture of more proximal tendons and consideration of the patient’s recovery and ultimate outcomes after hand surgery consultation.^2^,^3^

Considering pre-existing conditions is crucial in risk management assessments for hand interventions. Using our case as an example, the patient had RA. Rheumatoid arthritis increases the likelihood of both flexor and extensor tendon rupture due to recurrent episodes of tendon inflammation (tenosynovitis), tendon erosion, and malformed bone.^5^ The previous spontaneous abscess drainage or multiple antibiotics may explain the atypical presentation of the tendon itself, with it protruding from the abscess area likely due to the infection weakening the tendon.^3^,^4^ Additionally, looking at the patient’s leg, we saw that he suffered from severe PAD with the development acute right lower leg ischemia as well as left lower leg developing a stage 4 ulcer involving tendon and bone. This PAD in the lower extremities was also contributory to the upper extremity tendon rupture.

This case demonstrates that emergency physicians should take care when treating pathologies of the hand in patients with comorbidities such as RA, PAD, and CAD. Closed injuries such as sprains and extensor avulsions such as mallet finger can be splinted, but leaving exposed skin on a patient with known PAD and healing issues may lead to increased risk of rupture and subsequent complications. We suspect that if the outside hospital physician who saw the patient had performed incision and drainage of the hand without guidance from hand surgery, tendon injury may have inadvertently occurred. The outside hospital physician attempted to hospitalize the patient for antibiotics and further management; however, the patient refused admission and was discharged home.

A lesson to be taken from this case is to proceed with caution when intervening in the hand in the ED. Instrumentation for abscess drainage without proper imaging, consultation, and follow-up can lead to rupture and further complications. We recommend consideration of broad spectrum empiric antibiotic coverage for *Staphylococcus aureus* and Streptococcus species coverage^6^ (the most common causes of tenosynovitis, septic arthritis, and osteomyelitis) such as cefazolin or clindamycin with vancomycin added. For patients who will ultimately be discharged we recommend cephalexin or clindamycin. Lastly, one should always be cognizant of any comorbidities that a patient may have that can affect their ability to heal and or increase their risk for certain complications.

## CONCLUSION

In 2016 there were 42.3 million injury-related ED encounters nationwide, according to the National Center for Health Statistics.^3^ Of these, hand and upper extremity injuries accounted for 26%. While tendon injuries are not the most common, they can lead to lifelong disability if missed. This case illustrates the importance for emergency physicians to be aware of risk factors for tendon rupture, such as rheumatoid arthritis, and to tailor treatment accordingly. Tendon ruptures, partial or complete, that are open require emergent surgical intervention with wash out.^7^ This case highlights that one should use caution , with patients who do not have spontaneous rupture when intervening on the hand, due to the anatomy of tendons, muscles, and nerves that run superficially. Hand surgery is recommended in these cases due to complexity and high risk for poor outcomes.
